# Angioplasty and stenting for below the knee ulcers in diabetic patients: protocol for a systematic review

**DOI:** 10.1186/s13643-018-0897-0

**Published:** 2018-12-11

**Authors:** Carolina Dutra Queiroz Flumignan, Fábio Cabral Freitas Amaral, Ronald Luiz Gomes Flumignan, Vladimir Tonello Vasconcelos, Gabriela Araújo Attie, Raul Muffato Daolio, Henrique Jorge Guedes-Neto, Jorge Eduardo de Amorim, José Carlos Costa Baptista-Silva, Luis Carlos Uta Nakano

**Affiliations:** Division of Vascular and Endovascular Surgery, Department of Surgery, Escola Paulista de Medicina, Universidade Federal de São Paulo, Rua Borges Lagoa 754, São Paulo, 04038-001 SP Brazil

**Keywords:** Diabetic foot, Foot ulcer, Leg ulcer, Angioplasty, Endovascular procedures, Review, Evidence-based medicine, Meta-analysis

## Abstract

**Background:**

The worldwide incidence and prevalence of diabetes mellitus (DM) are increasing. DM has a high social and economic burden due to its complications and associated disorders. Peripheral arterial disease (PAD) is closely related to DM. More than 85% of patients with DM will develop PAD in their lifetime, and between 10 and 25% of patients with DM will have a foot ulcer. In such cases, it is important to determine for each patient whether it is necessary and feasible to revascularise the affected limb as well as the optimal technique. Percutaneous transluminal angioplasty (PTA) is designed to restore blood flow through the vessel lumen by various devices including balloons, drug-coated balloons, bare stents, drug-eluting stents and endovascular atherectomes. This systematic review aims to evaluate the effects of PTA in the treatment of lower limb arterial ulcers in diabetic patients.

**Methods:**

We will search randomised controlled trials (RCTs) and quasi-RCTs in the following databases (e.g., MEDLINE via PubMed, EMBASE, Lilacs, Cochrane Central Register of Controlled Trials, Ibecs, CINAHL, AMED, World Health Organization International Clinical Trials Registry Platform, ClinicalTrials.gov, and OpenGrey). Our search strategy will use the following free-text terms and controlled vocabulary (e.g., Emtree, MeSH) for ‘foot ulcer’, ‘leg ulcer’, ‘diabetic foot’, ‘Peripheral Arterial Disease’, ‘Diabetes Complications’, ‘Peripheral Vascular Diseases’, ‘critical limb ischemia’, ‘below the knee ulcer’, ‘angioplasty’, ‘stents’, ‘stenting’, and ‘endovascular procedures’. There will be no limits on date or language of publication. Two authors will, independently, select studies and assess the data from them. Risks of bias (RoB) of included studies will be evaluated using the Cochrane’s RoB tool. If possible, we will perform and report structured summaries of the included studies and meta-analyses. Results are not available as this is a protocol for a systematic review, and we are currently in the phase of building a sensitive search strategy.

**Discussion:**

While there are several available endovascular techniques for revascularisation, it is unclear which technique has better outcomes for ulcers below the knee in diabetic patients. A systematic review is required to validate and demonstrate these techniques and their outcomes to allow an evidence-based clinical decision.

**Systematic review registration:**

PROSPERO CRD42017065171

## Background

The worldwide incidence and prevalence of diabetes mellitus (DM) are increasing. In 2013, the USA spent over US$ 100 billion on the health care costs for diabetic patients [1, 2]. Ten to 25 % of all diabetic patients will develop a lower limb ulcer in their lifetime [3, 4]. An ulcer imposes significant limitations on the patient and requires a complex treatment plan to heal properly; these factors increase the economic and social burden of the disease. At least 50% of the patients with lower limb ulcer have an underlying peripheral arterial disease (PAD). In such cases, revascularisation of the limb may be required to restore blood flow to the distal limb [5, 6].

Revascularisation is indicated whenever a diabetic patient with a lower leg ulcer has any of the following characteristics: ankle-brachial index (ABI) < 0.8, ankle systolic pressure < 80 mmHg or a systolic toe pressure < 40 mmHg, or a transcutaneous oxygen tension (TcO2) < 40 mmHg and with at least one imaging exam demonstrating flow atherosclerotic lesion below the inguinal ligament: duplex ultrasound or angiography (by computed tomography, magnetic resonance imaging (MRI) or digital subtraction) [4].

Revascularisation can be achieved with a traditional open surgery bypass (using autologous vein or a prosthetic graft) or by endovascular techniques [7]. Endovascular revascularisation is a technique that aims to restore the blood flow through the arterial lumen using various devices. It can be performed by percutaneous transluminal angioplasty (PTA), subintimal angioplasty, intravascular stents, or atherectomy devices [8].

Currently, there is no substantial evidence to support that any of these techniques demonstrates increased efficacy relative to the others [7]. The alleged benefits of endovascular approaches are decreased recovery time, less pain at the surgical site, fewer complications as infection, and a lower total operational risk. Such endovascular approach would allow the elderly and patients with multiple comorbidities to be treated. Limitations of an endovascular approach include the high cost of the devices and medical apparel need to the procedures and the anatomical obstacles that might prevent passage of devices through the arterial lumen [7].

### Objectives

The purpose of this review is to assess the effects of endovascular revascularisation for arterial below the knee ulcers in diabetic patients. For the purpose of this systematic review, PTA will include all types of endovascular revascularisation techniques.

## Methods

This protocol is prospectively registered in PROSPERO database under number CRD42017065171. The Cochrane Handbook for Systematic Reviews of Interventions Reviews [9] was used to develop this protocol, and the Preferred Reporting Items for Systematic Review and Meta-Analysis (PRISMA-P) Protocols [10] were used to report it. The new Cochrane handbook version 5.2.0 (June 2017) and version 6 (September 2018) will be used in the final review, see the supplementary file 1 for the PRISMA-P checklist.

### Types of studies

For this systematic review, only randomised controlled trials (RCTs) and quasi-RCTs will be accepted.

### Types of participants

Inclusion criteria for patients with diabetes mellitus type 1 or type 2 include a presentation with leg ulceration below the knee and clinical and objective evidence of peripheral arterial disease. No limits on patient age, gender, or sex will be considered. We will consider a diagnosis of PAD when confirmed by a clinical exam demonstrating one or more of the following symptoms:

- Ankle systolic pressure (ASP) < 80 mmHg
- Ankle-brachial index (ABI) < 0.8
- Toe systolic pressure < 40 mmHg
- Transcutaneous oxygen tension (TcO2) < 40 mmHg

Diagnosis of PAD can be confirmed with at least one imaging technique (e.g., duplex ultrasound or angiography by computed tomography, MRI, or digital subtraction) demonstrating any stenosis below the inguinal ligament. The unit of analysis will be the participants, which will be considered in an intention-to-treat approach. For trials that consider multiple interventions in the same group, we will analyse only the partial data of interest. If there are any included studies that randomised parts of the body, each part will be treated separately for counting outcomes with the corresponding intervention group.

### Types of interventions

PTA will be compared as an intervention to either the best medical practice (BMP) alone or including a placebo or conventional surgery (e.g., autologous or prosthetic bypass). These same guidelines will apply to PTA used as adjunctive therapy and will also include a comparison to a different type of revascularisation where possible. Any dose of drug-eluting or drug-coated endovascular devices or any course duration of the intervention will be considered. Any studies that use PTA as the sole difference were considered.

### Types of outcome measures

#### Primary outcome

Our primary outcome will be healing of the largest ulcer or all ulcers below the knee including time-to-heal and ulcer recurrence data. Only total healing of the largest ulcer or all ulcers below the knee will be considered as treatment success; the reduction of the ulcer area should only be considered as favourable treatment in case of BMP. Healing outcomes will be assessed at 30 days, 6 months, 1 year, and annually from then onward. Trials using these time points will be grouped.

#### Secondary outcomes

The patients will be assessed for the following:

- Amputation rate which includes patients that underwent a major or minor amputation during the interval between intervention and the end of follow-up
- Adverse events will also be tracked to assess safety. Both severe adverse events (e.g., those immediately life-threatening or causing hospitalisation, incapacity, nerve injury, acute limb ischemia, or death) and mild to moderate adverse events (e.g., infection at the surgical site, complications at the access site, acute limb ischemia) will be tracked
- Primary or secondary vessel patency will be assessed by angiographic or ultrasonographic methods. The treated vessel will be analysed to ensure no flow obstruction after the first intervention (primary patency) or after subsequent interventions (secondary patency)
- Mortality defined as any cause of death directly related to cardiovascular events or infectious complications of the disease will also be tracked
- Quality of life, measured by any validated questionnaire (e.g., SF-36) or by related information such as time away from work
- Total length of hospitalisation

For the amputation rate, a limb loss of the heel and above defines the major amputation, such as Syme’s amputation, which requires a prosthetic device. Any tissue loss of the forefoot, such as a toe or trans metatarsal amputation, which does not require a prosthetic device, defines the minor amputation [6]. All definitions not mentioned in the text will be in accordance with international standards previous published [6, 11]. These outcomes will be assessed at the same time points as the primary outcome.

### Methods for search

#### Electronic searches

The search will be done at least in the following databases:

- Medical Literature Analysis and Retrieval System (MEDLINE via PubMed)
- Embase (via Elsevier)
- Cochrane Central Register of Controlled Trials - CENTRAL (via Wiley)
- Indice Bibliográfico Español de Ciencias de la Salud – IBECS (via Virtual Health Library)
- Latin American and Caribbean Center on Health Sciences Information – LILACS (via Virtual Health Library)
- Cumulative Index to Nursing and Allied Health Literature (CINAHL)
- The Allied and Complementary Medicine Database (AMED)
- World Health Organization International Clinical Trials Registry Platform (www.who.int/ictrp/en/)
- ClinicalTrials.gov (www.clinicaltrials.gov/)

The search strategy will consist of controlled terms (e.g., MesH and Emtree) and free-text terms related to ‘foot ulcer’, ‘leg ulcer’, ‘diabetic foot’, ‘peripheral arterial disease’, ‘diabetes complications’, ‘peripheral vascular diseases’, ‘critical limb ischemia’, ‘below the knee ulcer’, ‘angioplasty’, ‘stents’, ‘stenting’, and ‘endovascular procedures’. No limits for language, date or status of the publication will be used. A search will also be done in the grey literature source OpenGrey.eu, for identification of unpublished trials. The Table 1 show a MEDLINE via PubMed sample search strategy.

**Table 1:** MEDLINE PubMed search strategy

1	“Leg Ulcer”[Mesh] or (Leg Ulcer*) or (lower limb ulcer)
2	“Foot Ulcer”[Mesh] or (Foot Ulcer*) or (Plantar Ulcer*) or “below the knee ulcer”
3	“Diabetic Foot”[Mesh] or (Foot Diabetic) or (Diabetic Feet) or (Foot Ulcer Diabetic) or diabet*
4	“Peripheral Arterial Disease”[Mesh] or (Arter* Disease* Peripheral) or “critical limb ischemia”
5	“Diabetes Complications”[Mesh] or (Diabet* Complication*) or (Diabetes-Related Complication*) or (Diabetes Related Complication*) or (Complication* of Diabetes Mellitus) or (Diabetes Mellitus Complication*)
6	“Peripheral Vascular Diseases”[Mesh] or (Disease* Peripheral Vascular) or (Peripheral Angiopath*)
7	1 or 2 or 3 or 4 or 5 or 6
8	“Stents”[Mesh] or stent*
9	“Drug-Eluting Stents”[Mesh] or (Drug Eluting Stent*) or (Drug-Eluting Stent*) or (Drug-Coated Stent*) or (Drug Coated Stent*)
10	“Self Expandable Metallic Stents”[Mesh] or (Self Expandable Metal* Stent*)
11	“Angioplasty”[Mesh] or Angioplast* or (Endoluminal Repair*) or (Percutaneous Transluminal Angioplast*) or (Transluminal Angioplast*)
12	“Angioplasty, Balloon, Laser-Assisted”[Mesh] or (Laser Balloon Angioplast*) or (Laser-Assisted Angioplast*) or (Laser Assisted Angioplast*) or (Angioplast* Transluminal Percutaneous Laser) or (Laser-Assisted Balloon Angioplast*) or (Laser Assisted Balloon Angioplast*) or (Percutaneous Transluminal Laser Angioplast*)
13	“Angioplasty, Laser”[Mesh] or (Laser Angioplast*)
14	“Angioplasty, Balloon”[Mesh] or (Dilation* Transluminal Arter*) or (Balloon Angioplast*)
15	revascular* or (drug-coated balloon)
16	“Endovascular Procedures”[Mesh] or (Endovascular Procedure*) or (Intravascular Procedure*) or (Intravascular Technique*) or (Endovascular Technique*)
17	8 or 9 or 10 or 11 or 12 or 13 or 14 or 15 or 16
18	7 and 17
19	18 AND (Therapy/Broad[filter])

#### Hand search

Reference lists for all included studies, and review articles will be searched for additional trials. Manufacturers and specialists in the field of vascular surgery will be contacted for further research. Besides, the authors of the included studies will be contacted regarding additional data and other published or unpublished studies.

#### Selection of studies

Two independent reviewers (CDQF and RLGF) will evaluate all studies to determine if they are appropriate for inclusion. If there is any disagreement, a third reviewer (LCUN) will arbitrate or it will be solved by discussion with team authors.

Full-text articles will be used to determine the study’s appropriateness for inclusion. Excluded studies will be noted with specific exclusion criteria. Details of the selection process will be recorded, using the PRISMA flow diagram and table with the characteristics of excluded studies [10].

#### Data extraction and management

Data from included studies will be extracted by two independent reviewers (CDQF and RLGF). The following study characteristics and outcome data will be extracted [9]:

- Methods, including study design, the total duration of study and period of carryout, number and location of study centres, research setting, withdrawals, and date of study
- Participants, including total number, age aspects (e.g., mean, range), gender, the severity of the condition, diagnostic, and inclusion and exclusion criterion
- Interventions, including direct interventions, intervention comparison, concomitant medications, and excluded medications
- Outcomes, including planned and final primary and secondary outcomes and time points reported
- Notes, including trial funding for trial and notable conflicts of interest for trial authors.

One author (CDQF) will fill these data on Review Manager software (RevMan 5.3) for statistical analysis by two authors (CDQF and RLGF) [12].

#### Assessment of risk of bias in included studies

Risk of bias will be assessed by two independent reviewers (CDQF and RLGF) using Cochrane’s ‘Risk of bias’ (RoB) tool, described in Section 8.5 of the Cochrane Handbook for Systematic Reviews of Interventions [13]. These tools consist of (1) random sequence generation, (2) allocation concealment, (3) blinding of participants and personnel, (4) blinding of outcome assessment, (5) incomplete outcome data, (6) selective outcome reporting, and (7) other bias. Each domain must be graded as high risk, low risk, or unclear risk of bias according to the criteria described in the risk of bias table in the Cochrane Handbook [13]. Discussion with the authors’ team will resolve any RoB assessment conflicts. Blinding will be considered separately for different key outcomes when necessary. Treatment effects will be analysed in the context of the study’s risk of bias of hat contributed to the outcome. All risk of bias in included studies will be reported using the Revman 5.3 software [12].

#### Measures of treatment effect

Dichotomous data will be analysed as risk ratio. Continuous data will be analysed using mean difference for similarly scaled data. Continuous data with different scales will be compared using standardised mean difference. Confidence intervals of 95% will be used for all data.

#### Unit of analysis issues

Lower limbs will be the unit of analysis for all outcomes where possible, such as primary and secondary patency, amputation rate, and ulcer healing. The participant will be the unit of analysis for other outcomes including mortality, adverse events, quality of life, and length of hospitalisation. The intention-to-treat approach will be used.

#### Dealing with missing data

The authors or study sponsors of the included studies will be contacted where necessary to verify details about data characteristics or to provide missing numerical outcome data. Important bias is not expected if intervention groups have missing outcome data, and the reasons for missing data are balanced and reported across all groups. Missing data will be considered as significant bias in situations where the data gap will have different implications in the compared groups. Outcome frequency will influence the potential impact of missing data in dichotomous studies, while the frequency of participants missing data will impact continuous outcomes studies [9].

#### Assessment of heterogeneity

The studies will be assessed for methodological and clinical heterogeneity using the *I*^2^ statistic. Meta-analyses will be conducted if after these analyses the studies are considered homogeneous. We will analyse statistical heterogeneity by visual inspection of the forest plots where possible. While strict thresholds for the interpretation of *I*^2^ will not be applied, we will use the following Cochrane criteria to guide the heterogeneity interpretation [14]:

- 0 to 40%: might not be important
- 30 to 60%: may represent moderate heterogeneity
- 50 to 90%: may represent substantial heterogeneity
- 75 to 100%: considerable heterogeneity

The heterogeneity stratification demonstrates the percentage of variability in the estimated effects resulting from the heterogeneity rather than from sampling error [9]. In cases of substantial heterogeneity, subgroups within pre-specified groups will be analysed in an attempt to explain the heterogeneity. Publication bias findings may also be used.

#### Assessment of reporting biases

If there are greater than ten studies that need to be included in a meta-analysis, the presence of publication bias and other reporting bias will be assessed using funnel plots [9, 15, 16].

#### Data synthesis

Data will be synthesised using RevMan 5.3 software with a meta-analysis performed when feasible [12]. If there are not-important or moderate levels of heterogeneity, a fixed-effect model will be used. If there is substantial heterogeneity, a random-effect model will be used. In the event of considerable heterogeneity, we will not perform a meta-analysis, but the data will be described textually.

#### Subgroup analysis and investigation of heterogeneity

In the case of substantial or considerable heterogeneity, we will perform a subgroup analysis to explore possible causes. Subgroup analysis will be carried out for participants’ characteristics (age, gender, and race) and intervention characteristics (types of endovascular device, angiosome-oriented treatment, and targeting artery). We will attempt to contact the authors to obtain missing data when such data is not available from the original publications.

#### Sensitivity analysis

A sensitivity analysis will be conducted to determine the impact of the exclusion of studies with a high risk of overall bias. These additional analyses will be conducted excluding those studies with a high risk of bias in at least one of the main domains in the risk of bias tool (generation of randomisation sequence, allocation concealment, and blinding).

#### ‘Summary of findings’ table

We will use the GRADEpro software to generate a ‘Summary of Findings’ table for each outcome to be analysed in this review. Using the five Grading of Recommendations Assessment, Development, and Evaluation criteria (study limitations, consistency of effect, imprecision, indirectness, and publication bias), we will assess the quality of evidence in the meta-analyses of the pre-specified outcomes [17, 18]. These criteria will be judged using the methods and recommendations described in Section 8.5 and Chapter 12 of the Cochrane Handbook [9]. A table of containing and justifications for any departure from the GRADE analysis will be generated if necessary; the ‘Summary of Findings’ table will be made following the methods described in Chapter 11 and Chapter 12 of the Cochrane Handbook [9].

## Results

This is a prospectively registered protocol for a systematic review; results are not yet available. Currently, we are defining a search strategy and selecting studies for the review.

## Discussion

The increasing number of endovascular techniques to treat PAD leads us to believe that this may have improved their results along the time. However, there are no high-quality systematic reviews to confirm this assumption. Using the described protocol, we will attempt to generate a systematic review to identify and collate the best available evidence on the effects of endovascular revascularisation for the treatment of PAD in diabetic patients with lower limb ulcers.

## Abbreviations

GRADE: Grading quality of evidence and strength of recommendations for diagnostic tests and strategies
PRISMA: Preferred Reporting Items for Systematic Review and Meta-Analysis
RCTs: Randomised controlled trials
